# Shift of Neutrophils From Blood to Bone Marrow Upon Extensive Experimental Trauma Surgery

**DOI:** 10.3389/fimmu.2022.883863

**Published:** 2022-05-17

**Authors:** Michel P. J. Teuben, Marjolein Heeres, Taco Blokhuis, Roy Spijkerman, Eric Knot, Nienke Vrisekoop, Roman Pfeifer, Hans-Christoph Pape, Leo Koenderman, Luke P. H. Leenen

**Affiliations:** ^1^ Department of Trauma, University Medical Centre Utrecht, Utrecht, Netherlands; ^2^ Center for Translational Immunology, University Medical Centre Utrecht, Utrecht, Netherlands; ^3^ Department of Traumatology, University Hospital Zürich, Zürich, Switzerland; ^4^ Department of Surgery, Maastricht University Medical Center, Maastricht, Netherlands; ^5^ Department of Respiratory Medicine, University Medical Center Utrecht, Utrecht, Netherlands

**Keywords:** trauma, neutrophil, subsets, bone marrow, porcine model

## Abstract

**Introduction:**

Extensive trauma surgery evokes an immediate cellular immune response including altered circulatory neutrophil numbers. The concurrent bone marrow (BM) response however is currently unclear. We hypothesize that these BM changes include (1) a relative reduction of the bone marrow neutrophil fraction and (2) increasing heterogeneity of the bone marrow neutrophil pool due to (3) the appearance of aged/returning neutrophils from circulation into the BM-compartment.

**Materials and Methods:**

Eight pigs were included in a standardized extensive trauma surgery model. Blood and bone marrow samples were collected at baseline and after 3 hours of ongoing trauma surgery. Leukocyte and subtype counts and cell surface receptor expression levels were studied by flow cytometry.

**Results:**

All animals survived the interventions. A significant drop in circulating neutrophil counts from 9.3 to 3.2x10^6^ cells/ml (P=0.001) occurred after intervention, whereas circulatory neutrophil cell surface expression of CD11b increased. The concurrent bone marrow response included an increase of the BM neutrophil fraction from 63 ± 3 to 71 ± 3 percent (P<0.05). Simultaneously, the BM neutrophil pool became increasingly mature with a relative increase of a CXCR4^high^-neutrophil subtype that was virtually absent at baseline.

**Conclusion:**

The current study shows a shift in composition of the BM neutrophil pool during extensive trauma surgery that was associated with a relatively circulatory neutropenia. More specifically, under these conditions BM neutrophils were more mature than under homeostatic conditions and a CXCR4^high^-neutrophil subset became overrepresented possibly reflecting remigration of aged neutrophils to the BM. These findings may contribute to the development of novel interventions aimed to modify the trauma-induced immune response in the BM.

## Introduction

Trauma and subsequent surgery induce acute systemic inflammation and trigger activation of innate immune cells (1–3). Extensive ongoing trauma surgery is associated with an increased activation state of circulatory neutrophils and enhanced neutrophil migration to the tissue (4, 5). Furthermore, a prompt decline in circulatory neutrophil numbers upon insult has been described in specific cases (4–7) and linked to the development of inflammatory complications (4, 5). To maintain homeostasis of circulatory neutrophils, compensatory mobilization of cells originating from the bone marrow (BM) occurs. Consequently, drastic shifts in the constitution of the blood neutrophil pool during systemic inflammation has been reported (8, 9).

Enhanced mobilization of BM-neutrophils is considered to alter the content of the BM immune cell pool as well. To restore bone marrow immune reserves, enhanced hematopoiesis is mandated (10). Granulopoiesis is, however, considered a time-consuming process as maturation in the post-mitotic pool takes >4 days (11, 12). The compensation mechanisms in the BM and kinetics in response to increased cell demands are poorly understood (10, 13).

Profound inflammatory conditions have further been linked to the ´empty bone-marrow´ phenomenon (14, 15). This BM-condition is defined as a deficit of both post-mitotic neutrophils and their progenitors. An empty bone marrow has been reported at least 24 hours post-insult after trauma due to a mismatch between circulatory demand and bone marrow synthesis capacity (14–16). In addition to liberation of neutrophils from the bone marrow, remigration of relatively aged neutrophils from circulation into the bone marrow compartment has also been suggested (17, 18). The role of this phenomenon during a decrease in circulatory neutrophils during extensive surgery has not been studied before. To date, the exact interplay between changes of circulatory neutrophils after an acute trauma-induced inflammatory insult and the early bone marrow neutrophil-response has yet to be elucidated.

The hypothesis that is tested predicts that acute trauma-evoked depletion of systemic neutrophils is associated with altered composition of the bone marrow neutrophil pool. Such changes are characterized by (1) relative enhancement of the bone marrow neutrophil fraction and (2) increasing heterogeneity of the bone marrow neutrophil pool due to (3) the appearance of aged/returning neutrophils from circulation into the BM-compartment.

In order to test this hypothesis, a standardized porcine trauma model of extensive trauma surgery was utilized. The porcine model was chosen as this model allows for standardization of severe trauma and subsequent surgical interventions. Furthermore, porcine models have superior translational properties, as pigs are more closely related to humans in terms of their dimensions, anatomy, genetics, physiology and immunology compared to alternative non-primate animal models (19, 20).

## Materials and Methods

All experiments were performed in accordance with the guidelines of the Institutional Animal Care and Use. The study protocol was approved by the Saarland University Hospital Animal Care Committee.

### Experimental Animals

All experiments were performed as described in the application and *Female Landrace* pigs were utilized (50-60 kilograms).

### Experimental Procedure

Premedication included intramuscular midazolam (1mg/kg), ketamin (20mg/kg) and atropine (50ug/kg). Intubation was performed following 2 minutes of pre-oxygenation with 100 percent Oxygen at 10L/min. A volume-controlled ventilation protocol was utilized. Maintenance of anesthesia was achieved by Isoflurane 0,25-0,50%, continuous midazolam infusion (0,6 mg/kg/hour), and sufentanil infusion (15ug/kg/hour). Ventilation rates were guided by end tidal CO_2_-values and FiO_2_ of 0.3, Positive end-expiratory pressure of 0cm H_2_O and an I:E-ratio of 1:2 was preferred and aimed for in all animals. Frequent arterial blood gas analyses were performed to check ventilator and metabolic status. Antibiotics were not applied and hypothermia was prevented by altering room temperature. In accordance with the treatment concepts of the Definitive Surgical Trauma Care (DSTC™)-course (21), hypovolemia was corrected by additional sodium chloride 0.9% fluid resuscitation. Continuous arterial line blood pressure measurements were available. All animals were exposed to standardized extensive trauma surgery. The protocol has been described previously (22). In short, the following injuries were induced: liver laceration, 5 small bowel injuries, diaphragm injury, stomach, spleen, pancreas, left kidney. Furthermore, a thoracotomy was performed, and a cardiac injury was induced. Additionally, the left lung was injured and treated through hilar clamping and resection. Finally, the infrarenal inferior vena cava and right kidney were lacerated. All standardized injuries were treated by experienced trauma surgeons and anaesthetists and in line with treatment concepts of the DSTC™-protocols for trauma care (21). The sequence and execution of injury induction and surgical care were standardized and performed according to a standardized time-schedule. Animals were euthanized after 3 hours of ongoing surgery by pentobarbital infusion.

### Sampling

Blood and bone marrow samples were obtained at baseline and after 3 hours (immediately prior to euthanization). For blood neutrophil analysis, 9 mL of peripheral blood were collected from a central venous catheter in ethylenediaminetetraacetic acid (EDTA)-anticoagulated Vacutainers at baseline and directly after the animals were exposed to the final injury and subsequent therapy. An ice-cold isotonic NH_4_CL-lysis buffer was utilized twice for the lysis of erythrocytes and cells were washed in between with FACS-buffer (Dulbecco phosphate buffered saline supplemented with 2% heat inactivated fetal calf serum, 5mM EDTA). White blood cell numbers for staining have been standardized by the utilization of a Neubauer Chamber to calculate the appropriate dilution factor. Antibody mixes were added, and samples were incubated in dark conditions for 45 minutes on 4°C. Then, samples were washed twice with PBS2+ (phosphate-buffered saline with added sodium citrate [0.38% wt/vol] and pasteurized plasma proteins [10% vol/vol; Sanquin, Amsterdam, The Netherlands]. Following the final wash steps, cells were fixed with BD Cellfix (BD, Mountain View, CA, USA), a premixed fixing concentrate containing 1% formaldehyde and 0,1% sodium azide. Prior to the experiments the stability of all antibodies was tested and validated. All fixed samples were analyzed within 24 hours with a FACS Canto II flowcytometer (BD, Mountain View, CA, USA). Data from individual experiments are depicted as fluorescence intensity as the median fluorescence intensity (MFI). A minimum of 20,000 neutrophils per sample was analyzed. Populations not expressing the used markers were used to set background fluorescence levels and compensation matrixes were composed by using beads and the automated setup system for compensation in BD FACSDiva software version 6.1.3 (BD, Mountain View, CA, USA).

Bone marrow material was harvested in accordance with recommendations for humans (23, 24). In short, the pig was placed in the supine position with both legs fixed. The appropriate extremity was prepared with antiseptic, scrubbed and draped, only exposing the site to be sampled. A 1.5cm longitudinal skin incision was made and bone-covering soft tissue was removed. Thereafter a 2 mm unicortical entry point was drilled with a sterile hand drill at the anterior-medial aspect of the proximal tibial metaphysis (10cm distal from the knee joint). Thereafter, an EDTA-coated 50 milliliter syringe with 1ml and a 25-Gauge needle were used to aspirate BM-content from the cavity.

After collection, BM-samples were directly and simultaneously processed with corresponding blood samples. At baseline, material was collected from the left tibia, whereas after intervention cells were gathered from the right tibia. In our model no extremity trauma was applied.

### Flowcytometry Analysis of Porcine Peripheral Blood Samples

**Figure 1:**
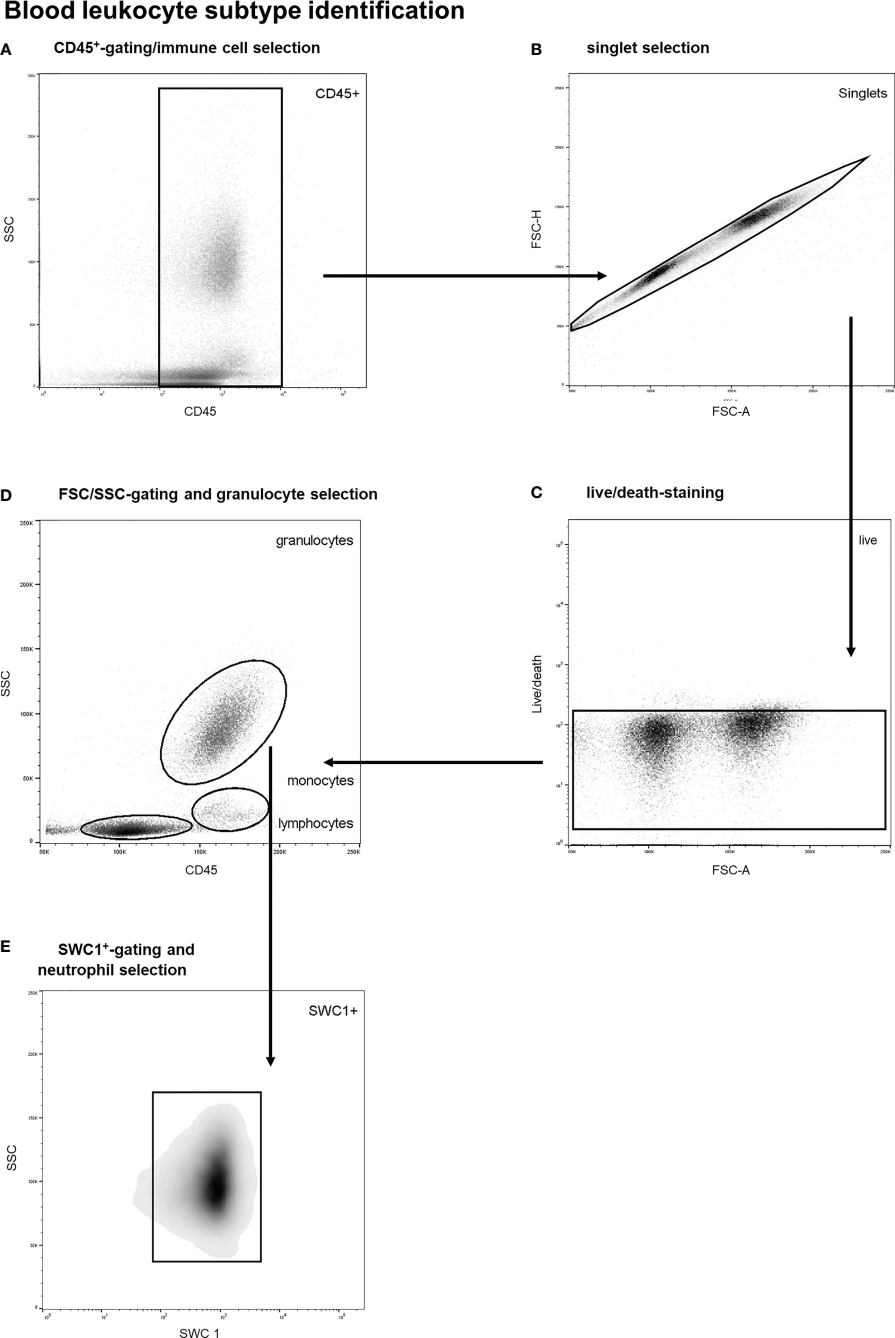
Stepwise neutrophil gating protocol as utilized for identification of circulatory leukocyte subtypes and neutrophils. **(A)** Selection of CD45 positive cells and thereby the exclusion of debris and non-immune cells. **(B)** Exclusion of doublets. **(C)** Life/death-stain and selection of viable immune cells. **(D)** Identification of different leukocyte subtypes and subsequent selection of granulocytes using FSC/SSC-plots. **(E)** Selection of SWC 1^+^ cells, and thereby exclusion of eosinophils. SSC, sideward scatter signal; FSC, forward scatter signal.

A previously validated gating strategy was applied to distinguish between circulatory leukocyte subtypes. First, nucleated, viable singlet leukocytes (CD45^+^-cells) were included (see **Figures 1A–C**). Thereafter, forward/sideward scatter (FSC/SSC) profiles were utilized to identify different leukocyte subtypes, as previously described (25, 26). Neutrophils were identified through typical high sideward scatter profiles as observed both in human and porcine blood samples (25–29). Furthermore, SWC 1 negative cells were excluded as this marker is expressed on porcine neutrophils and not on porcine eosinophils (29). A representative example of the gating strategy of blood leukocytes is shown in **Figure 1**. To obtain leukocyte populations for determination of reference values, monocytes as well as lymphocytes and blasts (pooled) were gated as well. CountBright counting beads (Invitrogen, Waltham, Massachusetts, USA) were utilized to count and compare absolute cell numbers over time.

### Determination of Bone Marrow Leukocyte Subtypes and the Neutrophil Fraction

**Figure 2:**
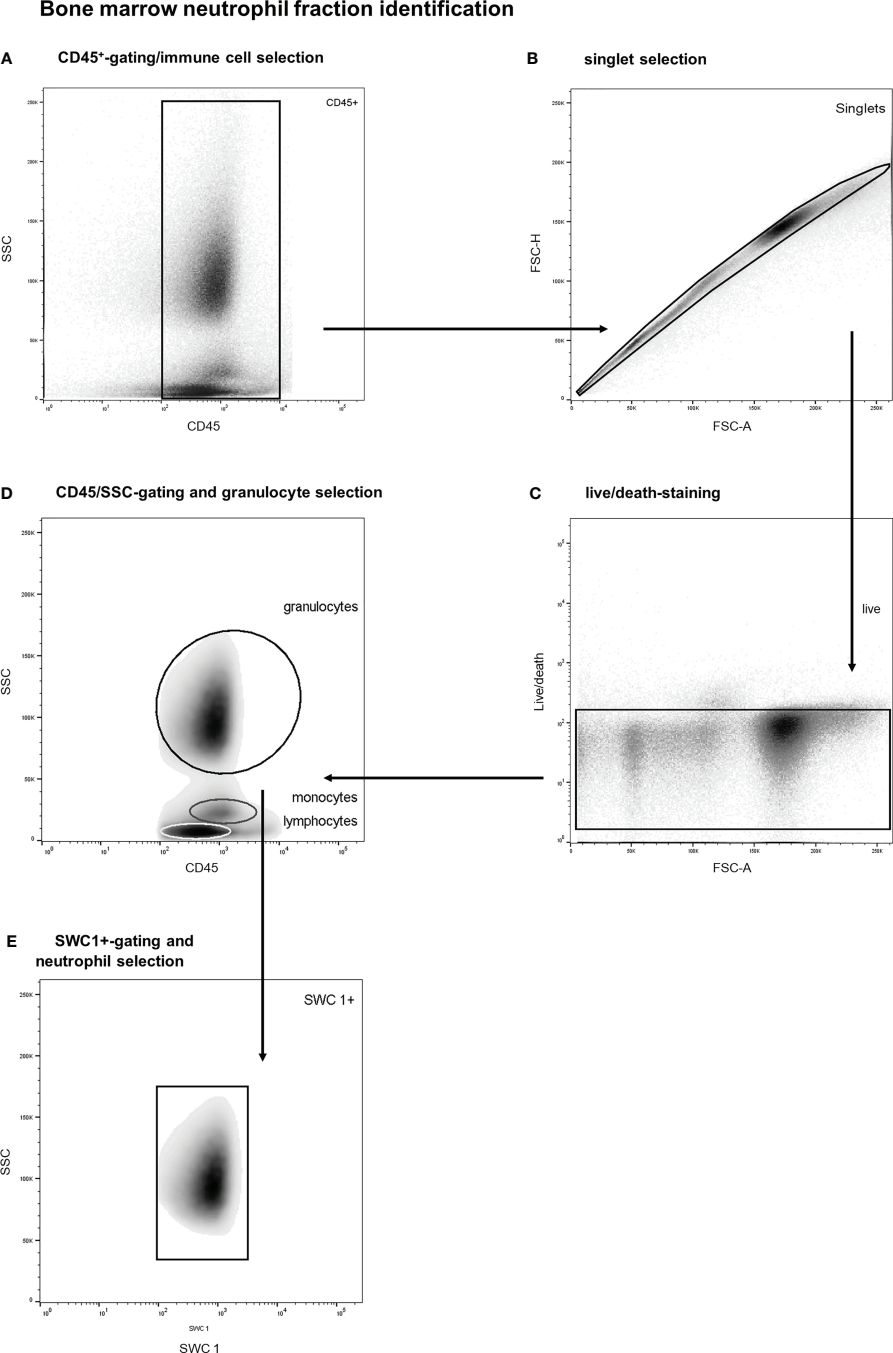
Stepwise neutrophil gating protocol as utilized for identification of the bone marrow neutrophil fraction. **(A)** Selection of CD45 positive cells and thereby the exclusion of debris and non-immune cells. **(B)** Exclusion of doublets. **(C)** Life/death-stain and selection of viable immune cells. **(D)** Identification of different leukocyte subtypes and subsequent selection of granulocytes (of note: basophils are not included in the granulocyte gate due to distinct side scatter signal). **(E)** Selection of SWC 1+ cells, and thereby exclusion of potential contamination by eosinophils. SSC, sideward scatter signal; FSC, forward scatter signal.

The bone marrow neutrophil fraction was defined as the proportion of marrow immune cells (CD45^+^) belonging to the neutrophil category, which was determined with flow studies (27–29). More specifically, bone marrow immune cells were identified by a multistep gating protocol. A representative example of this gating strategy is shown in **Figure 2**. First, vital BM-CD45^+^-cells were selected, and debris and doublets were excluded (see **Figures 2A–C**). Thereafter, a SSC/CD45 gate was used to select bone marrow granulocytes (see **Figure 2D**). Basophils were gated out based on their lower SSC signal. This is an established gating strategy for both porcine and human bone marrow analysis (27–29). Thereafter, SWC 1-positive cells were selected as this marker is exclusively expressed on porcine neutrophils and not on eosinophils (see **Figure 2E**) (29). This gating strategy has been validated by co-expression analysis of different leukocyte subtypes. Furthermore, validation experiments including cell sorting studies with morphologic analysis demonstrated a neutrophil purity over 99%. The BM-neutrophil fraction was determined by calculating the percentage of BM-neutrophils among all BM-immune cells.

### Monoclonal Antibodies and Flow Cytometry Measurements

For the flow cytometry analysis of neutrophil receptor expression, the following commercially available anti-pig monoclonal antibodies were obtained and when indicated conjugated by validated and commercially available antibody conjugation kits: SWC 1 (clone K263.3d7, Novus Biol, Centennial, Colorado, USA), SWC 3 (clone 74-22-15, Accurate Chemical, Westbury, New York, USA), SWC 8 (MIL-1, Abd Serotec, Kidlington, UK), CD11b (clone 2F4/11, Abd Serotec, Kidlington, UK), CD16 (clone G7, Abd Serotec, Kidlington, UK), CD29 (clone NaM160-1A3, BD, Mountain View, CA, USA), CD45 (clone K252.1E4, Abd Serotec, Kidlington, UK), CD45Ra (clone MIL-13, GenWay Biotech, San Diego, CA, USA), CD49D (clone L25, BD, Mountain View, CA, USA), CD184 (clone H-118, Santa Cruz Biotechnol, Santa Cruz, CA, USA). Lightning Link conjugation kits (Novus Biol, Centennial, Colorado, USA). A viability staining Vivid (Invitrogen, Waltham, USA) was added to exclude dead cells.

### Data Analysis and Statistics

Data was analyzed using software programs SPSS version 21.0 (SPSS Inc., Chicago, IL, USA), GraphPad Prism 8.0 (GraphPad Software Inc., La Jolla, CA, USA) and FlowJo Version 10 (*De Novo* Software, Glendale, CA, USA). Results were expressed as means (SEM) unless otherwise mentioned. For comparisons, Student’s T-tests, Paired T-testing* *or Mann Whitney U-tests were applied as appropriate. Equality of variance was tested using Lavene’s test. A *p*-value < 0.05 was considered statistically significant.

## Results

All subjects survived the interventions, and respiratory complications were not diagnosed. Bone marrow sampling was not successful in one experimental animal and, therefore, bone marrow analysis was not performed on cells from this animal. All other samples provided sufficient material for analysis.

### Decreased Circulatory Leukocyte Numbers and Altered Relative Presence of Leukocyte Subtypes Following Extensive Trauma Surgery

**Figure 3:**
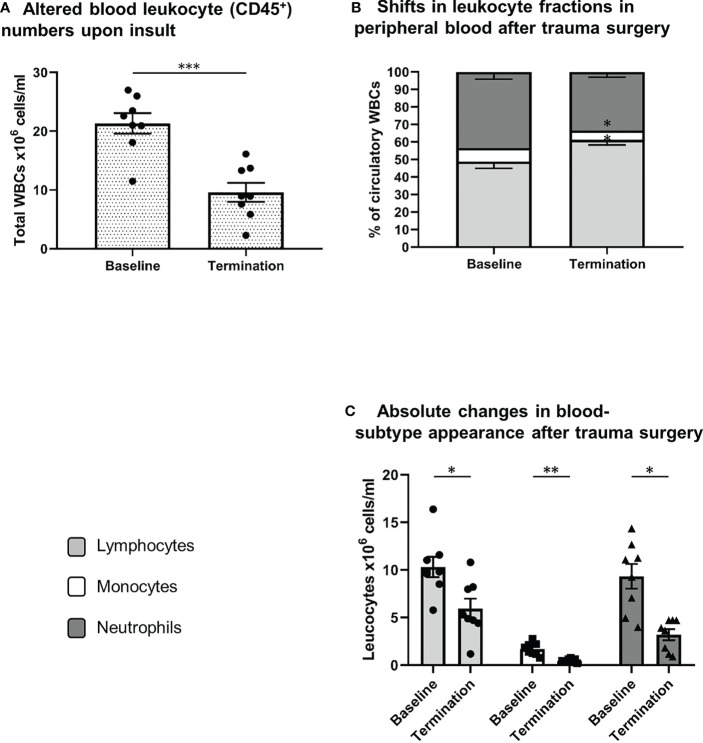
Systemic leukocyte alterations after trauma surgery. **(A)** Difference in white blood cell count between both conditions (baseline vs. termination). Relative **(B)** and absolute **(C)** differences in lymphocyte, monocyte and neutrophil numbers between baseline and termination, reflecting shifts in leukocyte fractions after trauma surgery. S/FSC, sideward/forward scatter signal; N, neutrophils; M, monocytes; L, lymphocytes *P < 0.05, **P < 0.01, ***P < 0.001. Baseline samples were taken prior to intervention and termination sampling was performed directly after the final procedure.

As shown in **Figure 3** extensive trauma-surgery is associated with a marked and statistically significant drop in leukocyte numbers in peripheral blood. At baseline a mean white blood cell count of 21.3 ± 1.7 x10^6^ cells/ml was measured, and after intervention leukocyte numbers reduced to 9.6 ± 1.6 x10^6^ cells/ml *(P<0.001)*. The identification of specific white blood cell subtypes is displayed in **Figure 1**. Absolute lymphocyte and neutrophil numbers both dropped significantly over time from 10.3 to 5.9 x10^6^ cells/ml *(P=0.01)*, and 9.3 to 3.2 x10^6^ cells/ml *(P=0.001*), respectively. The monocyte population decreased from 1.7 to 0.5 x10^6^ cells/ml (*P=0.01*; see **Figure 3**). The mean percentage of blood neutrophils as part of all leukocytes decreased from 43% to 33% *(P=0.07)*, whereas the percentage of blood lymphocytes increased significantly *(P=0.02).* This shows that extensive trauma surgery causes a shift in the constitution of the blood leukocyte pools characterized by decreased numbers of circulating immune cells and diminished neutrophil presence.

### Increase of the Bone Marrow Neutrophil Fraction During Trauma was Associated With a Relative Circulatory Neutropenia

**Figure 4:**
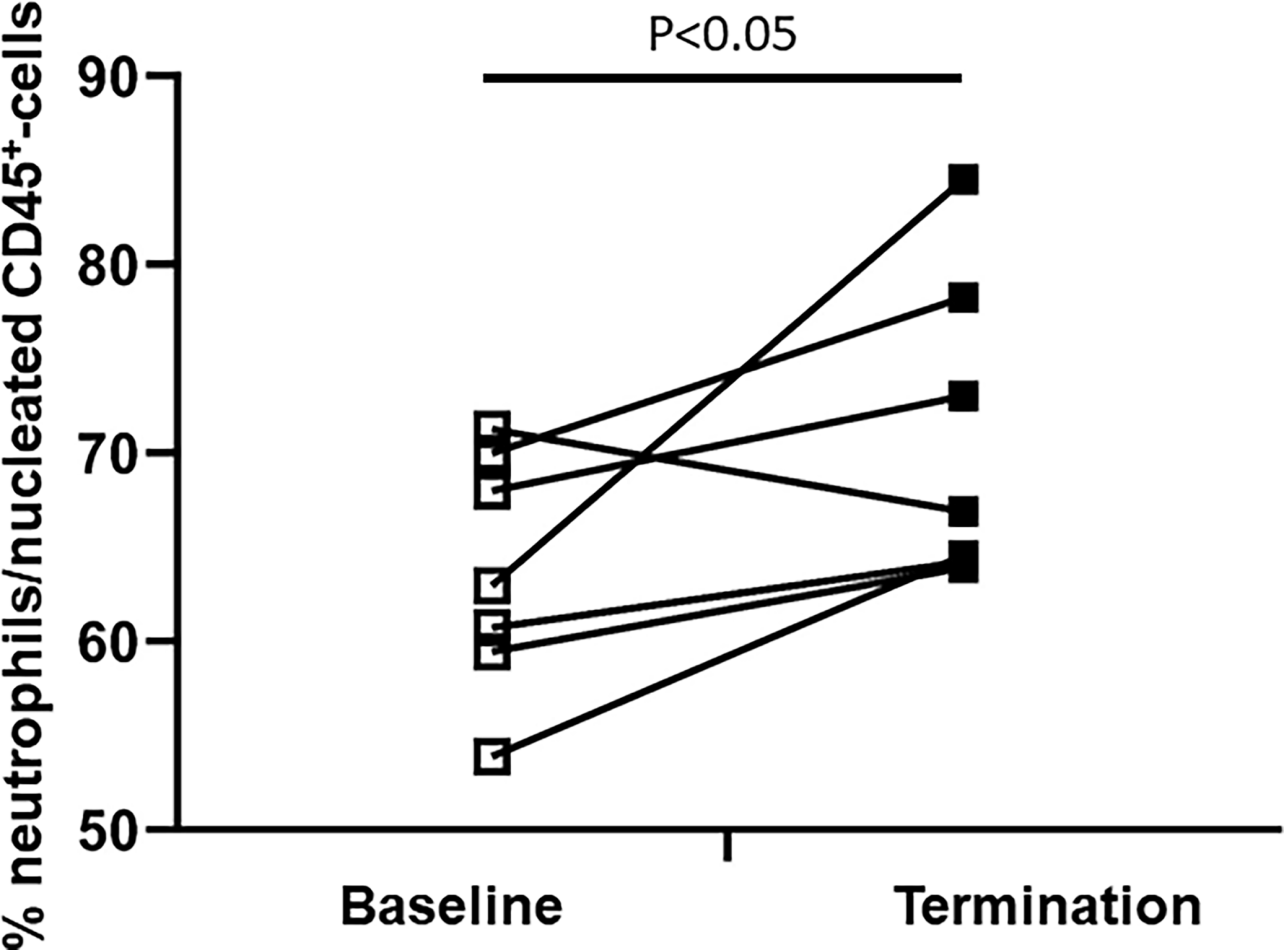
Difference in the bone marrow neutrophil fraction between baseline and termination. The BM-neutrophil pool/fraction has been identified as described in **Figure 2**.

The composition of the bone marrow neutrophil fraction changed strikingly after extensive trauma surgery. Porcine bone marrow neutrophils have been identified according to a multistep gating protocol as described in **Figure 2**. The neutrophil bone marrow fraction increased in 6 out of 7 experimental animals upon insult. Under homeostatic conditions, 63 ± 3 percent of CD45^+^ nucleated bone marrow cells were identified as neutrophils. However, following extensive surgery, this percentage increased up to 71 ± 3 percent (*P<0.05*, **Figure 4**).

### Dynamics in Expression of Cell Surface Receptors Reflect an Instantaneous Shift of Neutrophil Populations From Blood to Bone Marrow After Trauma Surgery

**Figure 5:**
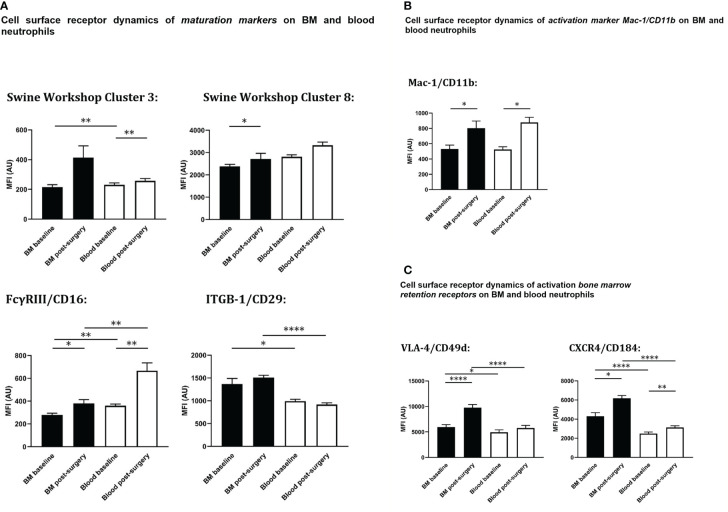
Cell surface expression on circulating and bone marrow neutrophils at baseline and after extensive surgery. **(A)** BM-maturation markers, **(B)** activation markers, **(C)** BM retention receptors. Black bars represent bone marrow samples, white bars represent blood samples. Baseline samples were taken prior to intervention and termination sampling was performed directly after the final procedure. Data are presented as mean ± SEM, MFI, median fluorescence intensity in arbitrary units. *P < 0.05; **P < 0.01; ****P < 0.001.

Next to a change in cell numbers of circulatory and bone marrow neutrophils, receptor expression profiles of neutrophils in both compartments changed as well. Changes were homogeneous across the different experimental animals as described in detail hereafter. Relevant receptors involved in porcine neutrophil maturation (**Figure 5A**), activation (**Figure 5B**) and bone marrow retention and homing (**Figure 5C**) were analyzed, using the previously described gating strategy (**Figure 1**) (blood samples) and 2 (bone marrow samples).

At baseline, the population of peripheral blood neutrophils had significantly higher neutrophil surface expression levels of both SWC 3 *(P<0.01)* and CD16 *(P<0.01)* compared with bone marrow neutrophils. After trauma, a statistically significant increase of both neutrophil SWC 3 *(P<0.001)* and CD16 *(P<0.001)* expression was found on circulatory neutrophils, whereas a less prominent increase of SWC 3 (statistically non-significant) and CD16-expression levels *(P<0.05)* was measured on BM-cells. As anticipated, levels of cell surface expression of CD29 (integrin β1-chain) were significantly higher on bone marrow neutrophils than on circulatory neutrophils. No differences were seen between homeostatic and post-insult conditions (**Figure 5A**).

As shown in **Figure 5B**, no statistically significant differences in cell surface expression levels of activation marker CD11b were seen between neutrophils from blood and BM. After extensive surgery, CD11b-expression on circulatory neutrophils significantly increased after insult *(P<0.05)*.

In addition, in homeostasis expression levels of CD184 (CXCR4) and CD49d (VLA4) involved in neutrophil retention in the bone marrow were statistically significantly lower on circulatory neutrophils compared to bone marrow *(P<0.001 and P<0.05, respectively)*. Following intervention, cell surface expression levels of CD184 *(P<0.05)* and CD49d *(P<0.001)* rose on bone marrow neutrophils. CD49d-expression on systemic neutrophils did not change after insult, whereas CD184-expression on circulatory neutrophils increased significantly *(P<0.01)* and VLA-4 on bone marrow neutrophils increased significantly after intervention. Cell surface receptor expression levels of CD49d and CXCR4 on blood and bone marrow neutrophils under different conditions are shown in **Figure 5C**.

### Increased Heterogeneity of the Bone Marrow Neutrophil Pool and Overrepresentation of an FCS^high^/CXCR4^high^-Neutrophil Subset Upon Extensive Porcine Trauma Surgery

As described previously, extensive trauma surgery is associated with striking changes in the cell-surface receptor expression profiles of the BM neutrophil pool (**Figures 5A–C**). Furthermore, the amount of variability of cell surface expression levels of relevant markers (**Figures 5A–C**) on BM neutrophils also increased after intervention, (**Figures 5A–C**), reflecting increased heterogeneity of the bone marrow neutrophil population after trauma.

**Figure 6:**
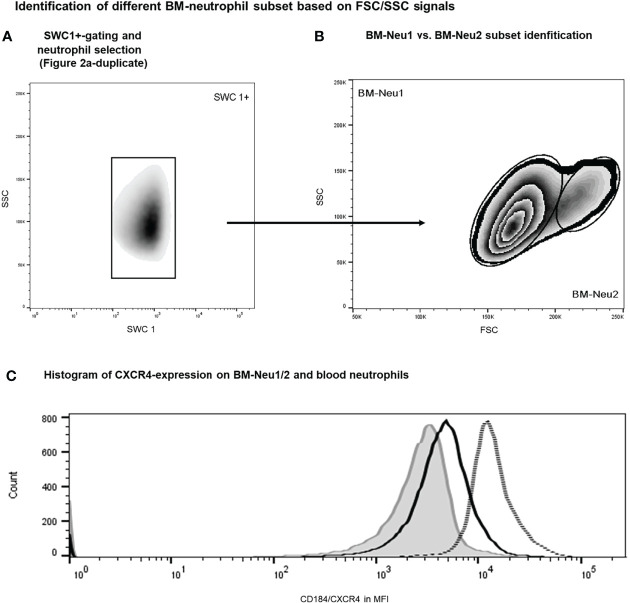
Gating protocol for the identification of BM-neutrophil subsets after intervention. Initial gating steps have been displayed in **Figure 2**. **(A, B)** Representative example of FACS analysis of porcine bone marrow samples after intervention. **(C)** Histograms showing regular neutrophils (BM-Neu1, black line), novel FSC^high^ neutrophils (BM-Neu2, grey line) and reference blood neutrophils at termination (grey filled curve). MFI, median fluorescence intensity.

Additionally, overrepresentation of a specific neutrophil subset of FSC^high^-neutrophils (BM-Neu2) after insult was demonstrated (**Figures 6A, B**). Forward and side scatter density plots of CD45^+^/SSC^high^/SWC 1^+^- cells (neutrophils) allow for identifying this specific subset (see **Figure 6B**). Under homeostatic conditions this subset (BM-Neu2) is virtually absent in bone marrow and represents 2.7 ± 0.2 percent of bone marrow neutrophils. However, after extensive trauma surgery, BM2-Neu-cells comprise 9.2 ± 1.0 percent of BM-neutrophils (**Figure 6B**). The relative increase of the size of the BM-Neu2 population as fraction of all BM-neutrophils after trauma was statistically significant *(P<0.001)*. BM2-Neu cells were further characterized by a statistically significant higher co-expression of CXCR4/CD184 *(P<0.01)*. A representative example of a histogram of CXCR4 expression on BM-Neu1 (regular neutrophils in BM), BM-Neu2 and a corresponding blood sample is displayed in **Figure 6C**. **Table 1** provides an overview of additional co-expression receptor analyses of both neutrophil subsets.

**Table 1:** Comparison of receptor co-expression profiles of BM-Neu1 and BM-Neu2 cells.

	BM-Neu1 (MFI)	BM-Neu2 (MFI)	P-value
SWC3	457 ± 68	356 ± 52	0.20
SWC8	2376 ± 205	2236 ± 806	0.07
FcγRIII/CD16	298 ± 45	385 ± 34	0.16
ITGB-1/CD29	1430 ± 164	902 ± 313	0.31
Mac-1/CD11b	859 ± 130	706 ± 70	0.52
VLA-4/CD49d	8497 ± 940	9777 ± 1156	0.70
CXCR4/CD184	5683 ± 530	14052 ± 1668	**0.0074**

Values in bold indicate statistically significant result. Data are presented as mean ± SEM. MFI, median fluorescence intensity in arbitrary units.

## Discussion

The key findings of the current study can be summarized as follows:

1. Extensive trauma-surgery in a standardized setting in pigs is associated with a prompt (<3hrs) decrease in circulating leukocyte numbers, including neutrophils.
2. Extensive trauma-surgery is associated with shifts in the composition of the bone marrow immune cell pools with an instantaneous relative increase of the BM-neutrophil fraction and an increased expression of neutrophil maturation markers on bone marrow neutrophils.
3. The post-traumatic BM-neutrophil pool is characterized by increased heterogeneity and overrepresentation of a unique CXCR4^high^ neutrophil subset.

Our study provides essential novel insights into the early cellular immune response to severe trauma surgery within the BM compartment. In line with other studies on extensive trauma surgery, an early increase of activation status (e.g. enhanced neutrophil CD11b/Mac-1 expression) and a systemic neutropenia were demonstrated following insult (30).

It has previously been demonstrated in clinical studies on surgical patients that aberrant circulatory neutrophil/leukocyte numbers after insult are linked to impaired clinical outcome (4, 5, 31–35). Both situations of early decreased circulatory neutrophil numbers (4, 5, 31) as reported in the current study, but also situations of early elevated systemic immune cell numbers (32–35), are associated with inferior clinical outcome. These observations imply that specific patterns of (divergent) circulatory neutrophil kinetics, with either reduced or increased cell numbers, may represent an abnormal cellular immune response. A large number of neutrophils and their progenitors are present in the bone marrow under tight control of their production, differentiation and eventually mobilization (8–10, 17). Therefore, it is key to understand the interplay between the bone marrow, peripheral blood and distant tissues. To our knowledge this is the first study that has investigated the bone marrow’s response to extensive trauma surgery and the related relative circulatory neutropenia in a controlled setting. Interestingly, and in contrast to the consensus (13, 14, 16), in the current study a relative increase of the BM-neutrophil fraction upon extensive surgical intervention was demonstrated, rather than depletion of the neutrophil bone marrow population (13, 14, 16).

The production and differentiation of granulocytes (granulopoiesis) mainly takes place in the bone marrow. Granulocytes and precursors make up 60% of BM-leukocytes (12). It is estimated that bone marrow produces approximately 0.5-1.0x 10^11^ neutrophils a day and that there are approximately 50-100 times more neutrophils in the bone marrow than in circulation (12, 36), but these issues are under current debate (37, 38). The bone marrow neutrophil population comprises of cells at different developmental stages. Three specific pools can be distinguished: the stem cell pool with self-renewal cell divisions, the mitotic pool (which includes cells (myeloblasts, (pro)myelocytes) that differentiate during proliferation) and the post-mitotic pool (with non-proliferating but maturing cells). The post-mitotic pool includes differentiated neutrophils (metamyelocytes, banded and mature cells) and forms a large bone marrow pool (39, 40). Mature neutrophils are released into circulation. After mobilization, neutrophils stay in circulation and may migrate into the tissue compartment before cells return to the bone marrow or other poorly defined tissue sites (17, 41). Under homeostatic conditions systemic neutrophil numbers remain constant due to a balance in production, compartmentalization, and cell death. However, in the case of acute systemic inflammation, neutrophil numbers in different compartments shift markedly (1–5, 42). Our research group has previously shown early neutropenia in a study on experimental extreme trauma surgery in pigs (22), which was reproduced in this study. Interestingly, these novel experiments reveal a simultaneous increase of the bone marrow neutrophil fraction. This is in contrast with several human studies in which the occurrence of an ´empty bone marrow phenomenon´ upon extensive surgery has been suggested (13, 14, 16). In these latter studies a marked decrease of the bone marrow neutrophil pool was seen after insult. This bone marrow neutropenia was explained by a putative exhaustion BM cell production. The differences between our results and these findings may partly be explained by differences in the timing of the different investigations. The current experiment focuses on the acute response to extensive surgery (first 3 hours) only, whereas other studies focus on later time-points (> 24 hours) (13, 14, 16). A similar early relative neutropenia followed by later neutrophilia has been described in a model of human experimental endotoxemia (43).

Besides affecting neutrophil numbers, extensive trauma surgery also led to profound changes in the characteristics of both blood and BM neutrophils. Upon intervention, circulatory neutrophil CD11b expression almost doubled. Similar effects have been described in severe trauma and reflect the marked effect of our extensive surgical model on systemic immune homeostasis (30, 44). In parallel to the relative neutrophilia of the bone marrow, characteristics of the BM-pool, determined by analysis of neutrophil surface expression profiles, changed as well after insult.

More specifically, cell surface expression levels of CD11b, CD16, CD184, SWC 8 on BM-neutrophils increased significantly after trauma-surgery. In addition, a non-statistically significant trend towards increased SWC 3 expression was observed as well after intervention. CD11b, CD16, CD184, SWC 3 and SWC 8 have been identified as maturation markers for bone marrow neutrophils, whose expression levels on neutrophils rise during maturation (29, 29, 45). Therefore, the findings from our study indicate increased overall maturation of the bone marrow neutrophil population (29, 46). Increased expression of CD184 on blood neutrophils after trauma may be secondary due to massive selective tissue migration of young cells under extreme inflammatory conditions. Alternatively, blood may function as a transport medium for tissue neutrophil returning back to the bone marrow. Of note, increased CD11b-expression on BM-neutrophils is most likely mainly due to increased cell activation (30, 44), rather than due to more progressed maturation. Increased bone marrow maturation after surgery can be explained by four processes that are not mutually exclusive:

Firstly, an increase in older neutrophils in the bone marrow might be caused by selective release of young cells into the circulation. This hypothesis is supported by the massive release of banded neutrophils in the first hours after severe trauma (22, 43).

Secondly, selective acceleration of processes of neutrophil proliferation, differentiation and maturation may occur upon extreme conditions; a situation generally coined as emergency granulopoiesis (47, 48). There are, however, no published studies supporting this phenomenon in trauma patients. Also, such emergency granulopoiesis after trauma would result in more immature, rather than aged neutrophils (47, 48). Thirdly, bone marrow neutrophil apoptosis may be affected or postponed after extensive trauma. As mentioned before, aged, but not necessarily apoptotic neutrophils are thought to be cleared in the bone marrow (17, 41). While bone marrow is thought to be involved in clearance of neutrophils, it is also known for its capacity to optimize cell survival by specific BM survival factors (49, 50). In cases of extensive trauma, regulation of cell survival in the bone marrow compartment may differ from regular conditions.

Lastly, enhanced selective influx of aged neutrophils into the bone marrow compartment may occur after trauma. Bone marrow homing of aged but not apoptotic neutrophils in murine models is thought to be a CXCR4 (CD184)-dependent process as cells can respond to stromal derived factor (SDF)-1alpha/CXCL12, the ligand for CXCR4 (45, 51). The bone marrow compartment constitutively expresses SDF-1alpha/CXCL12, and the BM is considered as the preferred homing compartment of CXCR4^high^-cells (18, 45). As such, increased BM accumulation of aged neutrophils, which have been trafficking back from circulation after extensive trauma surgery likely contribute to overall aging of the BM neutrophil population. Additionally, the current study is the first to describe prominent increase of a specific CXCR4^high^-neutrophil subset (termed BM-Neu2) in bone marrow after trauma. This increased population likely reflects returning neutrophils from circulation and has previously been described in non-trauma conditions as well (51, 52). The role and capacities of this subset are currently unclear.

### Limitations

Humoral factors upon standardized porcine trauma have been described in detail before and were not analyzed in the current study (53, 54). According to literature, systemic leukocyte neutrophil numbers in pigs range between 10 and 22 x10^9^/L and are higher than in humans (55). Baseline leukocyte numbers in the current study were within ranges as described in literature. Therefore, we do not believe that stress-induced neutrophilia due to transportation or handling of the pigs played a relevant role (56). Furthermore, as neutrophil subsets were identified after interpretation of the experiments, we were not able to perform *in vitro* studies on the novel BM-subset. For the current study we decided to utilize female subjects only. This should be considered when extrapolating of our findings to male trauma. Female animals were utilized as relevant hormonal fluctuations were unlikely to affect outcome in an extensive trauma study with a short observation period. Furthermore, it has been shown before in multiple species that utilization of female animals is not associated with increased variability of findings (57, 58). Moreover, housing of female animals is less challenging as they do not engage in hierarchy fights and female animals are easier to handle in the pre-operative phase than males. As a consequence, stress/cortisol-induced `baseline` alterations and subsequent increased variability were prevented (59, 60).

## Conclusion

This study describes the early bone marrow response to extensive trauma surgery in a controlled setting. The current study shows for the first time that during trauma-induced neutropenia, a parallel increase in neutrophil numbers in the bone marrow occurs. This shift is characterized by relative enrichment of the bone marrow neutrophil fraction, increased maturation-status of the bone marrow neutrophils, and an increased number of a specific CXCR4^high^-neutrophil subset in the bone marrow. This study also reveals that in pre-lethal trauma, aberrant neutrophil responses in blood and bone marrow go hand in hand. Hence, in order to design future immunomodulatory interventions for critically ill trauma patients it is important to acquire a better understanding of the pre-lethal bone marrow response. The porcine model is suitable to perform further studies on this issue and may also be utilized to perform future proof-of-principle interventions for pre-lethal trauma situations interventions.

## Data Availability Statement

The raw data supporting the conclusions of this article will be made available by the authors, without undue reservation.

## Ethics Statement

The animal study was approved by the Saarland University Hospital Animal Care Committee.

## Author Contributions

MT, TB, H-CP, LK and LL conceived and designed the project. MT, MH, RS, EK and NV performed the (pilot) experiments and initial data analysis. MT, TB, NV, RP, H-CP, LK and LL contributed to the analysis and the interpretation of the data. MT, NV, RP, H-CP, LK and LL wrote the first draft of the manuscript. MT, MH, TB, RS, EK, NV, RP, H-CP, LK and LL contributed to the final version of the manuscript. All authors contributed to the article and approved the submitted version.

## Funding

MT is supported by the Alexandre Suerman Stipend from the University Medical Center Utrecht.

## Conflict of Interest

The authors declare that the research was conducted in the absence of any commercial or financial relationships that could be construed as a potential conflict of interest.

## Publisher’s Note

All claims expressed in this article are solely those of the authors and do not necessarily represent those of their affiliated organizations, or those of the publisher, the editors and the reviewers. Any product that may be evaluated in this article, or claim that may be made by its manufacturer, is not guaranteed or endorsed by the publisher.
